# Health of Criminal Women

**Published:** 1882-12

**Authors:** 


### HEALTH OF CRIMINAL WOMEN.

Dr. M. M. Mosher, in an article upon this subject (*Boston Medical and Surgical Journal*), comes to the following conclusions :

*First.*—Intemperance and unchastity are the two vices which fill our penal institutions with women.

*Second.*—The influence of these vices is detrimental to health of body, increasing its susceptibility to disease, and lessening its recuperative power.

*Third.*—The diseases which follow as a direct result of these vices are syphilis, alcoholism, dyspepsia, rheumatism, and general anæmia.

*Fourth.*—Morbid conditions of the body react upon the moral nature, increasing and perpetuating the tendency to criminality; hence the importance of careful medical supervision as a reformatory measure.

*Fifth.*—More ample provisions should be made in all large cities for the isolation and through treatment of venereal patients of *both sexes*, either by the addition of special wards to the general hospitals or by the establishment of hospitals for this class.

*Sixth.*—The women who commit high crimes, that is, larceny, burglary, arson, manslaughter, etc., possess a more sensitive nervous organization than those who commit only offences against chastity and public order.—*Medical Record.*

